# A Case of a Young Patient With Necrotizing Soft Tissue Infection Caused by Community-Acquired Methicillin-Resistant Staphylococcus aureus

**DOI:** 10.7759/cureus.91877

**Published:** 2025-09-09

**Authors:** Ryuhei Igeta, Reina Sasaki, Tetsuya Matsumoto, Hiroshi Kaneko, Hidemasa Nakaminami, Shunya Ikeda, Takashi Shiga

**Affiliations:** 1 Department of Public Health, Graduate School of Medicine, International University of Health and Welfare, Chiba, JPN; 2 Department of Emergency Medicine, International University of Health and Welfare Narita Hospital, Chiba, JPN; 3 Department of Plastic and Reconstructive Surgery, International University of Health and Welfare Narita Hospital, Chiba, JPN; 4 Department of Infectious Diseases, International University of Health and Welfare Narita Hospital, Chiba, JPN; 5 Department of Clinical Microbiology, Tokyo University of Pharmacy and Life Sciences, Tokyo, JPN

**Keywords:** community-acquired methicillin-resistant staphylococcus aureus (ca-mrsa), necrotizing soft tissue infection (nsti), negative-pressure wound therapy, panton–valentine leukocidin (pvl)-associated s. aureus infection, split thickness skin graft, usa300

## Abstract

Necrotizing soft tissue infections (NSTIs) are rare but life-threatening conditions characterized by rapidly progressive tissue destruction. Although methicillin-resistant *Staphylococcus aureus* (MRSA) is a well-known pathogen, community-acquired MRSA (CA-MRSA) is an uncommon cause of NSTI, especially in otherwise healthy pediatric patients.

We report the case of a previously healthy 13-year-old boy who developed NSTI of the lower extremity after sustaining a trivial insect bite. On admission, he presented with rapidly progressive swelling, erythema, and severe pain out of proportion to physical findings. Computed tomography revealed fat stranding and thickening of the superficial fascia, and the finger test was positive. Emergency debridement revealed a necrotic area of subcutaneous fat and fascia measuring approximately 15 × 8 cm, and MRSA was identified in swab cultures. The patient required re-debridement, followed by negative pressure wound therapy (NPWT). A split-thickness skin graft was performed on day 21, after confirming negative wound cultures, with no graft-related complications. The patient was discharged in good condition after 62 days of hospitalization.

This case illustrates that CA-MRSA, though rarely causing NSTI, can lead to severe, rapidly progressive infections even in healthy children. Clinicians should maintain vigilance, as even minor injuries may serve as portals of entry. Early recognition, timely surgical debridement, and empiric MRSA coverage are critical to optimizing outcomes, and this case contributes to the limited literature on pediatric CA-MRSA NSTI in Japan.

## Introduction

Necrotizing soft tissue infection (NSTI) is a rare but life-threatening infection characterized by rapid destruction of subcutaneous tissue and fascia. Early diagnosis can be challenging because the initial symptoms are often nonspecific, yet prompt recognition and surgical intervention are crucial for survival [1].

Community-acquired methicillin-resistant Staphylococcus aureus (CA-MRSA) has emerged as an important public health concern, as it can cause serious infections in otherwise healthy individuals outside of healthcare settings [2,3]. Although CA-MRSA most commonly causes skin and soft tissue infections (SSTIs), it can occasionally lead to severe invasive diseases such as bacteremia, pneumonia, or, rarely, NSTI [2,4]. Panton-Valentine leukocidin (PVL)-positive strains such as USA300 have been increasingly recognized for their high virulence and association with severe infections [3,5].

While CA-MRSA is a less common etiology of NSTI, the global incidence has been steadily increasing [2]. The increased incidence has also been reported in the United States, where 14 out of 843 patients with MRSA in wound cultures developed NSTI [6]. Japan has also been seeing rising rates of CA-MRSA, particularly PVL-positive USA300, as recent surveillance studies demonstrated [3,5]. However, only one published case of CA-MRSA NSTI has been reported in Japan [7].

This report aims to present a rare case of CA-MRSA NSTI in a healthy child and to emphasize its significance for clinical practice. This case presentation provides clinical and microbiological findings that exemplify the broader epidemiological concerns introduced above.

## Case presentation

A healthy 13-year-old Japanese male baseball player presented with right lower leg swelling five days after a potential insect bite to his knees. He had initially visited a local doctor the previous day and was prescribed antibiotics (cefaclor) and an ointment (tetracycline hydrochloride). Due to worsening pain, high fever, and difficulty ambulating, he was transferred to our hospital.

**Figure 1:**
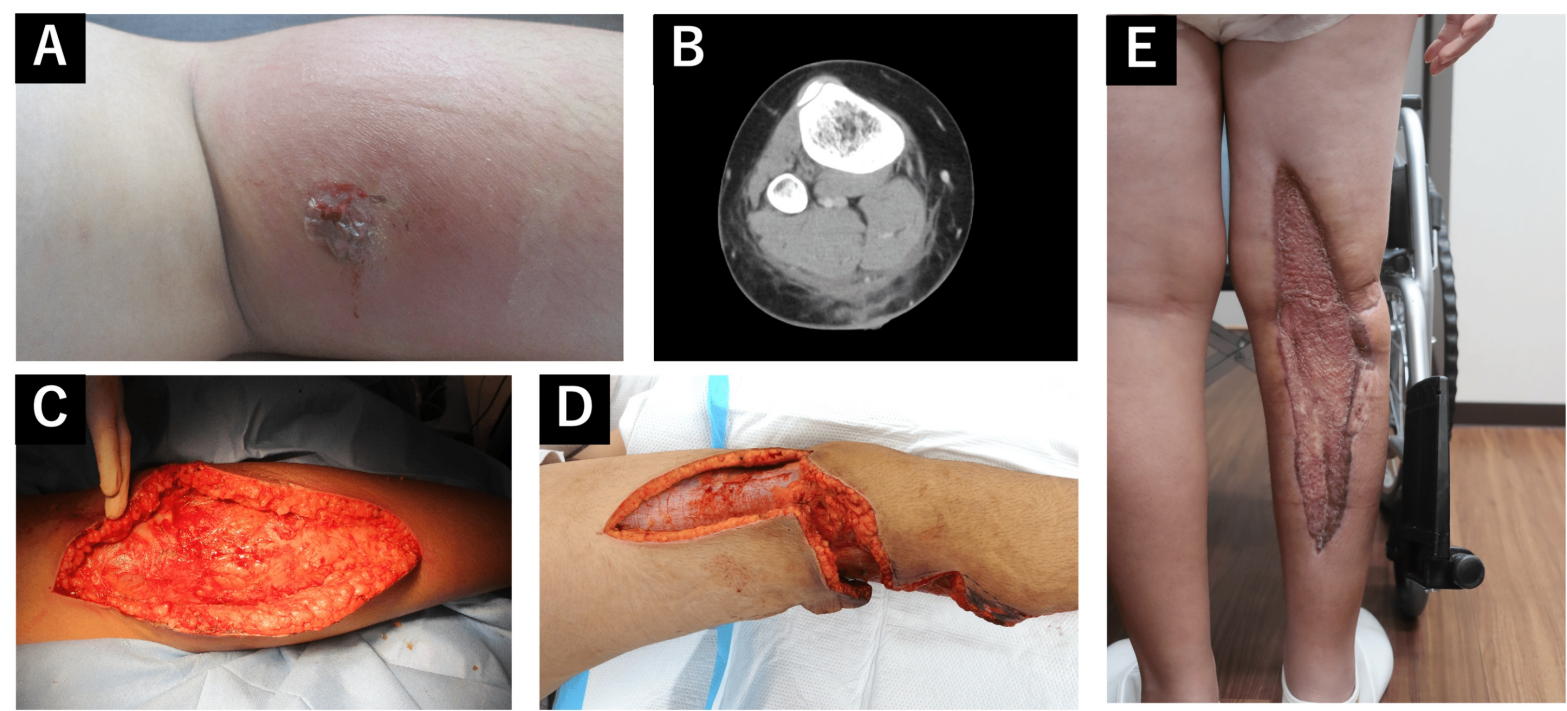
Clinical features of patient A: The patient’s right lower extremity on admission, showing swelling and erythema around the insect bite lesion located 5 cm distal to the popliteal fossa. B: Contrast-enhanced computed tomography (CT) scan of the right lower extremity demonstrating fat stranding and thickening of the superficial fascia (arrows), which are characteristic findings of necrotizing soft tissue infection. C: Intraoperative photograph of the wound bed after extensive surgical debridement, revealing necrotic fascia and subcutaneous tissue. D: An additional incision over the lateral femoral area. E: the wound after split-thickness skin grafting on day 21, demonstrating adequate graft take and progressive wound closure.

On arrival, the patient presented with rapidly progressive swelling, erythema, and severe pain out of proportion to physical findings, which limited movement of the affected limb. At presentation, the patient’s vital signs were as follows: body temperature of 37.2℃; pulse rate of 88 beats per minute; blood pressure of 141/76mmHg; respiratory rate of 18 breaths per minute; oxygen saturation (SpO2) of 98% on room air. A bite mark was found 5 cm distal to the right popliteal fossa, with swelling and pain extending from the posterior right thigh to the external leg (Figure 1A).

Laboratory data revealed an elevated leukocyte count (12,340/μL), mild microcytic anemia (hemoglobin - 12.4 g/dL, mean corpuscular volume, 81.4 fL), C-reactive protein (CRP) level of 3.05 mg/dL, and a lactate acid level of 0.8mmol/L (Table 1).

**Table 1:** Laboratory findings

Parameter	Patient values	Reference range
Leukocyte count	12,340/μL	4,000-9,000/μL
Hemoglobin	12.4g/dL	13.5-18.0g/dL
Mean corpuscular volume	81.4fL	85-100fL
C-reactive protein	3.05mg/dL	<0.3mg/dL
Lactate acid	0.8mmol/L	0.5-1.5mmol/L

No signs of liver, kidney, or coagulation dysfunction were observed based on laboratory tests. Contrast-enhanced computed tomography (CT) revealed cloudy fatty tissue and thickened superficial fascia extending from the right ankle to the knee, with increased density around the popliteal artery (Figure 1B). The finger test result was positive with foul odor, and the area was locally incised, suggesting NSTI. The finger test is performed by making a small incision down to the deep fascia and inserting a gloved finger to assess the ease of blunt dissection between tissue planes; minimal resistance and foul odor indicate a positive result. Emergency debridement revealed a necrotic area of subcutaneous fat and fascia measuring approximately 15 × 8 cm (Figure 1C), and an odor was detected in the same area. MRSA was detected in swab cultures from the same site; however, the blood culture results were negative. Bacterial identification and antimicrobial susceptibility testing were performed using the MicroScan WalkAway 96 Plus system (Beckman Coulter, Brea, CA, USA). In addition, MALDI-TOF (Matrix-Assisted Laser Desorption/Ionization Time-of-Flight) mass spectrometry (Bruker, Bremen, Germany) was employed for rapid identification in blood culture samples. Antimicrobial susceptibility results were interpreted according to Clinical and Laboratory Standards Institute (CLSI) guidelines [8]. The antimicrobial susceptibility testing results for the isolated MRSA are shown in Table 2.

**Table 2:** Antimicrobial susceptibility testing MIC: minimum inhibitory concentration; R: resistant; S: susceptible

Antibiotics	MIC (µg/mL)	Result
Penicillin G	>8	R
Oxacillin	>2	R
Ampicillin	>8	R
Sulbactam/ampicillin	16	R
Cefazolin	<8	R
Cefoxitin	>4	R
Imipenem/cilastatin	<1	R
Gentamicin	<2	S
Arbekacin	<1	S
Minocycline	<2	S
Clindamycin	<0.5	S
I-clindamycin	<4	S
Erythromycin	>4	R
Levofloxacin	>4	R
Vancomycin	1	S
Teicoplanin	<2	S
Daptomycin	<0.25	S
Fosfomycin	<4	S
Mupirocin	<256	S
Linezolid	2	S
Sulfamethoxazole-trimethoprim	<1	S
Rifampicin	<0.5	S

**Figure 2:**
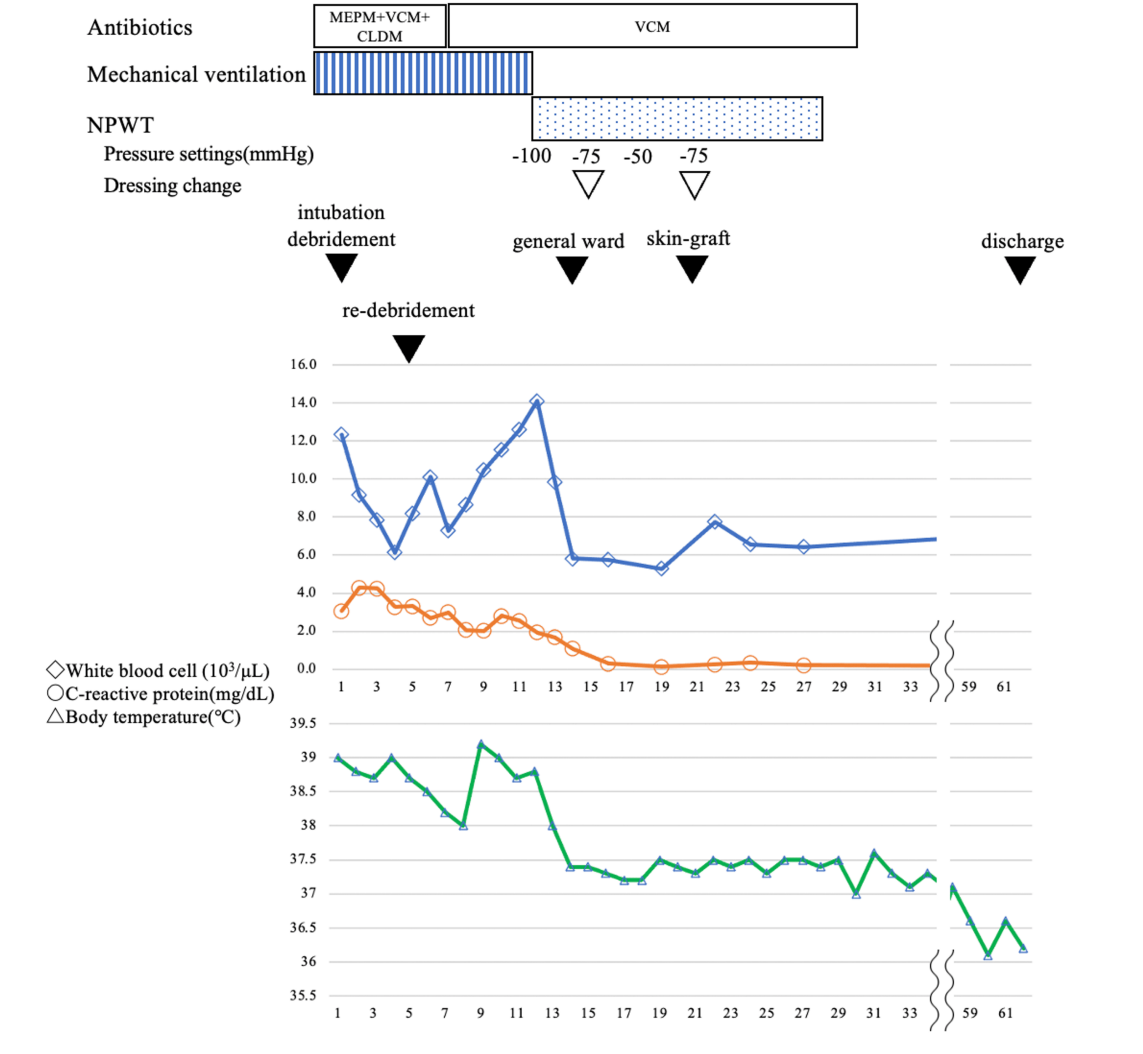
Clinical course and treatment after hospitalization The upper graph shows the white blood cell count (103/μL) and C-reactive protein level (mg/dL) as the blood test data. The lower graph shows the change in body temperature (℃). Timeline of antibiotics, mechanical ventilation, NPWT, and key clinical events (debridement, re-debridement, skin grafting, discharge) with corresponding changes in WBC, CRP, and body temperature. NPWT: negative pressure wound therapy; WBC: white blood cell (WBC); CRP: C-reactive protein (CRP); MEPM: meropenem; VCM: vancomycin; CLDM: clindamycin

Daily wound washouts were performed, and antibacterial agents (meropenem, vancomycin, and clindamycin) were administered after admission to the intensive care unit (ICU). On day 4, the inflammation had worsened, necessitating a further incision (Figure 1D). On day 10, negative pressure wound therapy (NPWT) was initiated, and on day 12, the patient was extubated. On day 14, the patient was transferred from the ICU to the general ward. On day 21, delayed primary closure with a split-thickness skin graft was performed, followed by post-operative epithelialization. We confirmed that the wound culture was negative before the skin grafting procedure. Although the patient initially experienced restricted knee joint mobility postoperatively, rehabilitation was undertaken, leading to gradual improvement. At the time of discharge, the patient was able to ambulate independently without assistance, and no stricture was observed. Subsequently, the patient was discharged on day 62 in good condition (Figure 1E). The patient’s clinical course and treatment after hospitalization are shown in Figure 2.

After discussion with the plastic surgery service, vancomycin was discontinued on day 30.

Subsequent analysis of MRSA revealed that it was staphylococcal cassette chromosome mec type ΨIV, positive for PVL, arginine catabolic mobile element type I, and sequence type 8 by multilocus sequence typing, which is the major PVL-positive MRSA clone, ΨUSA300, in Japan [3].

## Discussion

CA-MRSA, occurring outside healthcare settings, remains susceptible to non-β-lactam antibiotics but has high virulence, occasionally causing serious infections in healthy individuals [5,9]. CA-MRSA commonly causes skin and soft tissue infections (SSTIs) and rarely causes NSTIs [2]. Epidemiologically, the prevalence of PVL-positive CA-MRSA strains, particularly USA300, has been increasing both globally and in Japan [2,3]. Recent surveillance studies have demonstrated a significant rise in such strains in community settings, underscoring their growing public health importance [5,7]. In Japan, PVL-positive CA-MRSA cases, especially ΨUSA300, have been increasing [3,5]. PVL is a toxin comprising two components that are produced by *Staphylococcus aureus* and is related to SSTI [10]. Recent surveillance studies have demonstrated a significant rise in such strains in community settings, underscoring their growing public health importance [3,5]. However, reports of necrotizing soft tissue infection (NSTI) caused by CA-MRSA remain rare, especially in pediatric populations [4].

Our case involved PVL-positive ΨUSA300, likely triggered by a minor skin injury following a suspected insect bite. The exact entry route is unclear, but both the bite and the scratching could have allowed bacterial invasion. Similar infections from minor injuries have been reported [7,11].

PVL-positive *Staphylococcus aureus* infections in children commonly present as bacteremia, pneumonia, or osteoarticular infections [4]. NSTI from insect bites is rare, making this case notable. The severe course (ICU stay, surgery, prolonged hospitalization) reflects the virulence of ΨUSA300 and the need for caution, even in healthy children.

The reasons for NSTI progression remain unclear but may involve bacterial virulence, host susceptibility, minor trauma, and delayed diagnosis. Host factors like immune response and timing of treatment could influence severity. In children with stable vital signs, the decision for debridement should be guided by clinical findings rather than hemodynamic status alone. In our case, despite stable vitals, debridement was performed based on severe pain, a positive finger test, and intraoperative necrosis. The procedure was limited to non-viable tissue to minimize functional and cosmetic sequelae.

In contrast, a U.S. series found no mortality in CA-MRSA NSTI cases, though complications like prolonged ICU stays and reconstructive surgery were common [6]. In general, early recognition, prompt surgical intervention, and empiric antibiotic coverage for MRSA are critical to improving outcomes in patients with necrotizing soft tissue infections [1]. Our patient required long-term rehabilitation.

PVL-positive *Staphylococcus aureus* activates the p38 MAPK pathway, inducing IL-8 release and localized inflammation before tissue necrosis occurs [12]. This may explain the relatively low CRP level in this case despite NSTI. PVL-positive MRSA infections may show atypical inflammatory markers, requiring careful assessment. As 29% of U.S. cases occur in healthy individuals [6], empirical anti-MRSA therapy should be considered, even without risk factors [7,11].

This case also illustrates a practical clinical message. While definitive diagnosis of NSTI requires imaging and surgical assessment, there are key “red flags” that should raise suspicion even for non-specialists: a minor injury followed by rapidly worsening swelling, severe pain disproportionate to physical findings, and progressive functional limitation. Emphasizing these early warning signs may help clinicians recognize similar cases more promptly and guide timely referral and intervention.

## Conclusions

In conclusion, we present a rare case of NSTI caused by CA-MRSA in a healthy child following an insect bite. This case demonstrates that even trivial injuries can lead to severe infections due to PVL-positive strains such as ΨUSA300. Clinicians should be aware of this possibility and maintain vigilance, especially as CA-MRSA prevalence is rising in Japan and globally. In general, early recognition, prompt surgical debridement, and empiric MRSA coverage remain essential to optimize outcomes. This case also contributes to the limited literature on pediatric CA-MRSA NSTI in Japan, underscoring its epidemiological and clinical relevance.

## References

[1] McDermott J, Kao LS, Keeley JA, Grigorian A, Neville A, de Virgilio C (2024). Necrotizing soft tissue infections: a review. JAMA Surg.

[2] DeLeo FR, Otto M, Kreiswirth BN, Chambers HF (2010). Community-associated meticillin-resistant Staphylococcus aureus. Lancet.

[3] Kaneko H, Kanai M, Saito T (2023). Significant increase in the prevalence of Panton-Valentine leukocidin-positive methicillin-resistant Staphylococcus aureus, particularly the USA300 variant ΨUSA300, in the Japanese community. Microbiol Spectr.

[4] Garbo V, Venuti L, Boncori G (2024). Severe Panton-Valentine-leukocidin-positive Staphylococcus aureus infections in pediatric age: a case report and a literature review. Antibiotics (Basel).

[5] Yamaguchi T, Nakamura I, Sato T (2022). Changes in the genotypic characteristics of community-acquired methicillin-resistant Staphylococcus aureus collected in 244 medical facilities in Japan between 2010 and 2018: a nationwide surveillance. Microbiol Spectr.

[6] Miller LG, Perdreau-Remington F, Rieg G (2005). Necrotizing fasciitis caused by community-associated methicillin-resistant Staphylococcus aureus in Los Angeles. N Engl J Med.

[7] Arakawa S, Uchikawa R, Miyagawa A, Kaneko H, Nakaminami H, Nishimoto S (2023). The first case of necrotizing fasciitis caused by Panton-Valentine leukocidin-positive methicillin-resistant Staphylococcus aureus USA300 clone in Japan. J Dermatol.

[8] Clinical and Laboratory Standards Institute (CLSI) (2025). CLSI M100: Performance Standards for Antimicrobial Susceptibility Testing, 35th ed. https://clsi.org/shop/standards/m100/.

[9] Klevens RM, Morrison MA, Nadle J (2007). Invasive methicillin-resistant Staphylococcus aureus infections in the United States. JAMA.

[10] Shallcross LJ, Fragaszy E, Johnson AM, Hayward AC (2013). The role of the Panton-Valentine leucocidin toxin in staphylococcal disease: a systematic review and meta-analysis. Lancet Infect Dis.

[11] Paulis J, Tay ET (2018). Necrotizing fasciitis with pulmonary septic emboli following an infected insect bite. Am J Emerg Med.

[12] Yoong P, Pier GB (2012). Immune-activating properties of Panton-Valentine leukocidin improve the outcome in a model of methicillin-resistant Staphylococcus aureus pneumonia. Infect Immun.

